# Asymmetric response to ranibizumab in mixed choroidal neovascularization in a neovascular age-related macular degeneration diagnosed on OCT angiography – case report

**DOI:** 10.1186/s12886-021-01810-z

**Published:** 2021-01-15

**Authors:** Martin Pencak, Miroslav Veith

**Affiliations:** Department of Ophthalmology, Third Faculty of Medicine, Charles University, Prague and University Hospital Kralovske Vinohrady, Srobarova 1150/50, Prague, 100 34 Czech Republic

**Keywords:** Anti-VEGF, Mixed CNV, Age‐related macular degeneration, Resistance, Case report

## Abstract

**Background:**

To present a case report of a patient with a mixed choroidal neovascular membrane (CNV) with an asymmetric response to ranibizumab diagnosed on optical coherence tomography angiography (OCTa).

**Case presentation:**

A 61-year-old male was referred to our department in September 2017 due to decreased vision in his left eye. Best-corrected visual acuity (BCVA) was 43 Early Treatment Diabetic Retinopathy Study (ETDRS) letters in the left eye. Macular edema was present in the left eye, and a mixed CNV was identified on the OCTa. Therapy with intravitreal ranibizumab was commenced. After 5 ranibizumab injections, the BCVA was 42 ETDRS letters, and considerable intraretinal edema was still present. OCTa showed a resolution of the type 2 lesion of the mixed CNV; however, the type 1 lesion had continued to grow. The patient was then switched to intravitreal aflibercept. After 3 monthly aflibercept injections, the BCVA improved to 53 ETDRS letters, and a reduction of the edema was observed on the optical coherence tomography (OCT). OCTa showed a decrease in both the area and vessel density in the type 1 lesion of the CNV. Therapy with aflibercept was continued; however, while the intraretinal edema continued to improve, atrophy developed in the macula and the BCVA worsened to 43 ETDRS letters.

**Conclusions:**

Ranibizumab nonresponse in a neovascular age-related macular degeneration is not uncommon. However, to our knowledge, this is the first described case of an asymmetric response to ranibizumab in a mixed CNV. While the type 2 lesion of the CNV reacted swiftly to the ranibizumab therapy, the type 1 lesion continued to grow. As with some other cases of ranibizumab resistance, switching to aflibercept proved effective.

## Background

The neovascular form of age-related macular degeneration (nAMD) is a multifactorial chronic degenerative disease affecting the macular area of retina [1]. It is characterized by the presence of choroidal neovascular membrane (CNV) in the macula. Intravitreal anti-vascular endothelial growth factor (anti-VEGF) injections are currently used in the treatment of the disease and are usually able to stop disease progression and improve visual acuity in most nAMD patients [2–5]. There is, however, a group of patients who do not respond well to this treatment, either from the very beginning or later during the course of the treatment [6, 7]. The exact reasons for this phenomenon are not known. There is evidence showing favorable outcomes after switching from one anti-VEGF agent to another in these patients [8, 9]. In this case report, we present a patient with a mixed CNV where an asymmetric response to ranibizumab was observed in each portion of the CNV.

## Case presentation

**Fig. 1:**
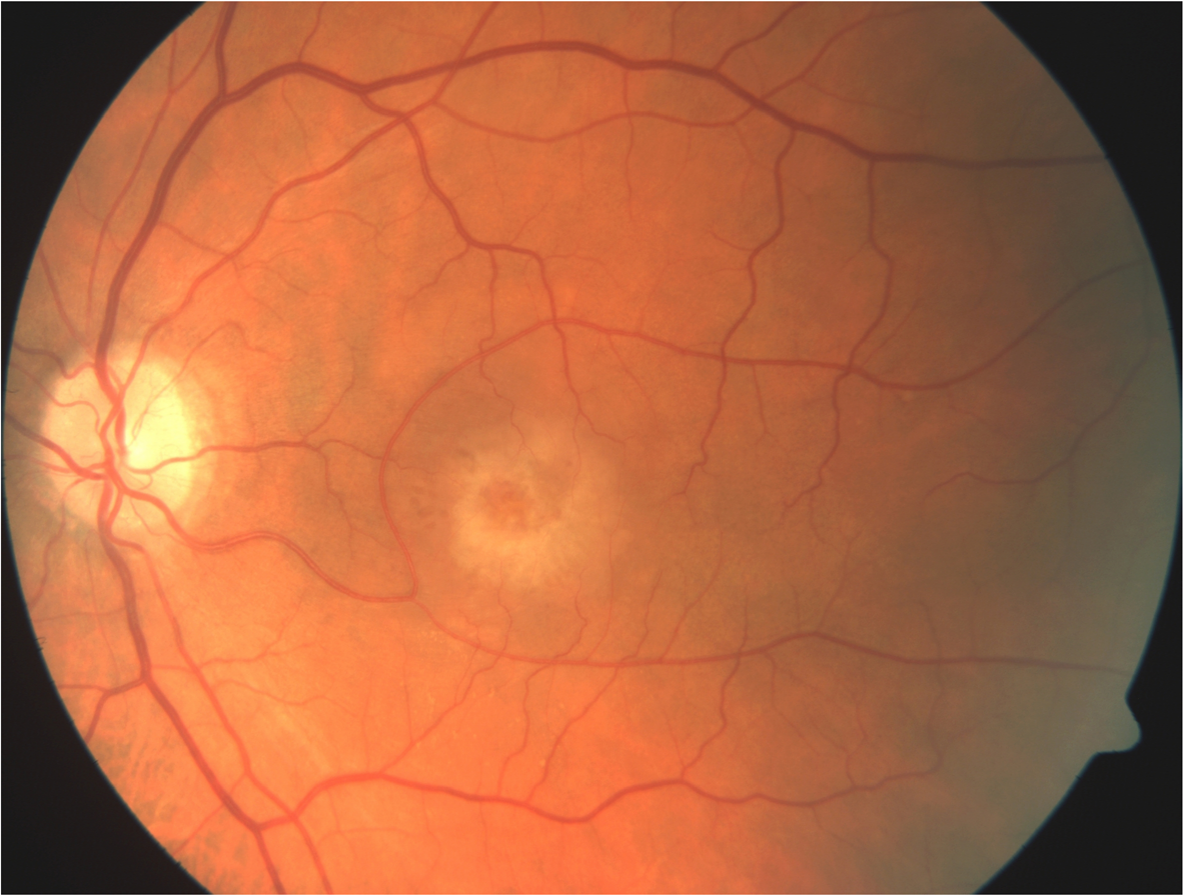
Color fundus photograph of the left eye showing a circular greyish lesion and edema in the foveal region

**Fig. 2:**
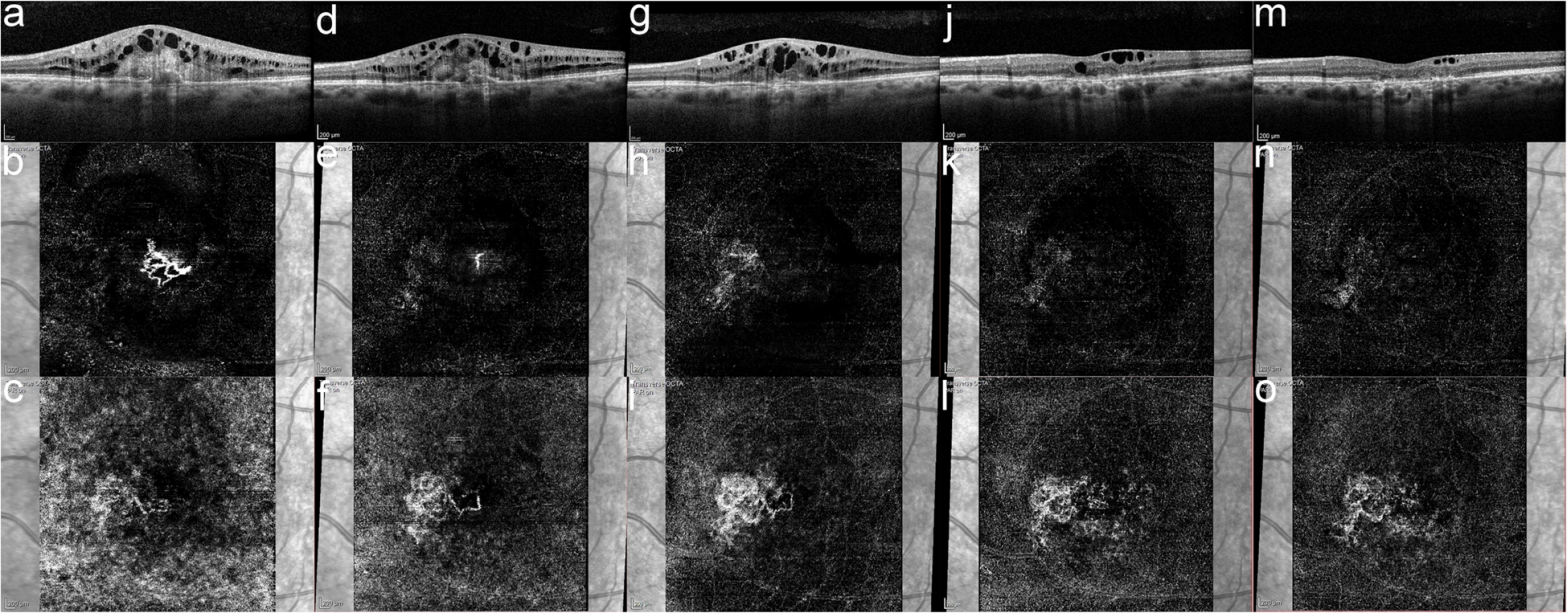
OCT and OCTa of the left eye, **a** baseline OCT - a dense lesion above a small reflective pigment epithelial detachment and intraretinal cystic edema surrounding the lesion, **b** baseline OCTa above RPE - type 2 lesion of the mixed CNV, **c** baseline OCTa below RPE - type 1 lesion of the mixed CNV, **d** OCT after 3 ranibizumab injections - some reduction of the dense lesion can be observed, however the intraretinal edema is still present, **e** OCTa above the RPE after 3 ranibizumab injections - just a residual feeder vessel remains, **f** OCTa below the RPE after 3 ranibizumab injections - increase in both the area and density of the type 1 lesion of the mixed CNV, **g** OCT after 5 ranibizumab injections – persistent intraretinal edema, **h** OCTa above the RPE after 5 ranibizumab injections – type 2 lesion of the mixed CNV is completely absorbed, **i** OCTa below the RPE after 5 ranibizumab injections – further growth of the type 1 lesion of the mixed CNV, **j** OCT after 3 aflibercept injections - reduction in edema, some intraretinal cysts are still present, **k** OCTa above the RPE after 3 aflibercept injections – no visible CNV, **l** OCTa below the RPE after 3 aflibercept injections - reduction in both the area and vessel density in the type 1 lesion of the mixed CNV, **m** OCT after 7 aflibercept injections - some intraretinal cysts still present in the macula along with atrophy of both the RPE and the outer retinal layers, **n** OCTa above the RPE after 7 aflibercept injections – no visible CNV, **l** OCTa below the RPE after 7 aflibercept injections – further reduction in both the area and vessel density in the type 1 lesion of the mixed CNV

A 61-year-old male was referred to our department in September 2017 due to decreased vision in his left eye (LE) over the preceding 6 months. His ocular history was negative, and his medical history included arterial hypertension, hypercholesterolemia, and a cardiac stent implanted 4 years prior. The patient was on antiplatelet therapy with acetylsalicylic acid. The best-corrected visual acuity (BCVA) was 80 Early Treatment Diabetic Retinopathy Study (ETDRS) letters in the right eye (RE) and 43 ETDRS letters in the LE. Intraocular pressure was within normal limits in both eyes. A slit-lamp examination of the anterior segment was physiological in both eyes. Fundus biomicroscopy showed drusen in the macula of both eyes and a circular greyish lesion and edema in the foveal region of the LE (Fig. 1). Optical coherence tomography (OCT) was performed, showing a dense lesion above a small reflective pigment epithelial detachment (PED) and intraretinal cystic edema surrounding the lesion (Fig. 2a). The central subfield thickness (CST) was 719 µm. OCT angiography (OCTa) showed a type 2 CNV above the retinal pigment epithelium (RPE) in the subretinal space (Fig. 2b). Under the RPE, a poorly circumscribed type 1 CNV was visible with a feeder vessel connecting it to the type 2 CNV above (Fig. 2c). Because the CNV was clearly visible on the OCTa, fluorescein angiography was not performed. A diagnosis of nAMD with a mixed CNV was made and therapy with intravitreal ranibizumab was introduced starting with the initial dose of 1 injection per month for 3 months. After the 3 injections, the BCVA in the LE improved slightly to 49 ETDRS letters. However, OCT showed persistent intraretinal cystic edema in the macula, and the CST was 647 µm. Flattening of the PED was observed as was the absorption of the dense lesion above the RPE (Fig. 2d). OCTa indicated a resolution of the type 2 CNV above the RPE, where only the feeder vessel remained visible (Fig. 2e). In the subRPE space, the type 1 CNV was not only present but had greater vascular density than before the introduction of ranibizumab (Fig. 2f). The therapy with ranibizumab was continued and the patient received 2 more monthly injections. On the follow-up visit 7 months after the baseline visit and after 5 ranibizumab injections, BCVA in the LE was 42 ETDRS letters, OCT showed cystic edema, and the CST was 610 µm (Fig. 2g). The OCTa of the area above the RPE showed that the feeder vessel had disappeared, and there was no sign of CNV (Fig. 2h). However, both the area and vessel density of the type 1 CNV in the subRPE space had increased (Fig. 2i). Due to the non-response of the type 1 lesion of the CNV to the ranibizumab, the therapy was switched to aflibercept. The patient received 3 monthly injections of aflibercept. Two months after the last aflibercept injection, the BCVA had improved to 53 ETDRS letters. The CST was 334 µm and a reduction of the edema was observed on the OCT (Fig. 2j), although some persistent intraretinal cysts were still present. OCTa showed stable findings in the space above the RPE (Fig. 2k). A reduction in both the area and vessel density of CNV in the subRPE space was observed (Fig. 2l). Therapy with aflibercept was continued using a fixed regimen of 4 bimonthly injections. In June 2019, one month after the 7th aflibercept injection, the BCVA worsened to 43 ETDRS letters and the CST was 273 µm (Fig. 2m). OCTa findings above the RPE remained stable (Fig. 2n), below the RPE there was a further reduction in both the area and vessel density of the type 1 lesion of the CNV (Fig. 2o). Some intraretinal cysts were still present in the macula along with atrophy of both the RPE and outer retinal layers. The patient received another 2 injections of aflibercept without any effect on the BCVA or intraretinal cysts. Treatment was then discontinued due to the lack of efficacy, i.e., presence of atrophy in the macula. No adverse events related to intravitreal injection procedure, ranibizumab nor aflibercept were recorded during the patient follow-up.

## Discussion and conclusions

In our patient, we observed an asymmetric response to ranibizumab in each portion of a mixed type 1 and type 2 CNV. The type 2 CNV responded swiftly with the closing of the network of small-caliber vessels with just a single feeder vessel remaining visible. However, the type 1 lesion of the mixed CNV continued to grow both in size and vessel density despite intensive treatment with ranibizumab. Kim et al. described a different response to anti-VEGF treatment in type 1 and type 2 CNV [10]. The type 1 CNV showed no reduction in size after anti-VEGF treatment, whereas the type 2 CNV showed a significant decrease in lesion size. They observed no change in vessel density in either group after anti-VEGF treatment. The difference in BCVA improvement was not significant between the two groups. In our patient, we observed a similar difference in the change of lesion size between the two parts of the mixed CNV after the anti-VEGF treatment; however, the vessel density actually increased in the type 1 part of the mixed CNV and high persistent edema was present in the macula despite intensive treatment with ranibizumab. Therefore, we believe that in our patient, the type 1 part of the CNV was unresponsive to ranibizumab. Nonresponse to various anti-VEGF drugs has been described in the past and switching to a different anti-VEGF agent has been shown to be effective in some nonresponders [6, 9, 11].

The reasons for the different responses to treatment in individual patients are not clear. It is probably a combination of several factors. Response to treatment can be influenced by a patient’s genetic profile [12]. In patients treated with ranibizumab and bevacizumab, the presence of neutralizing antibodies against these drugs has been recorded [4, 13]. However, the role of antibodies in the nonresponse to treatment with these drugs is not clear. Response to treatment may also be influenced by the development of alternative angiogenesis signaling cascades in the presence of VEGF blockade or by neovascular membrane maturation with the migration of pericytes to new blood vessels [14]. The difference in ranibizumab and aflibercept distribution in the retina was previously described in monkey eyes [15]. Ranibizumab permeates the retina via intercellular clefts, while aflibercept concentrates in ganglion cells, cells of the inner and outer retinal layers, and the RPE. Both drugs enter the intraretinal and choroidal vessels. However, it is not clear whether this difference in the distribution has any clinical significance.

It is hard to tell which of these factors may have influenced the asymmetric response in our patient. However, we believe that any anomaly in the genetic profile of the patient or the presence of neutralizing antibodies against ranibizumab would have affected both parts of the CNV. With regard to the development of alternative angiogenesis signaling cascades, we believe that this would have taken some time and number of ranibizumab injection to develop, but the type 1 CNV was nonresponsive right from the start of treatment with ranibizumab. We could not find any study comparing the intraocular levels of different vascular growth factors between various CNV types. It is possible that in our patient, the development of the type 1 part of the mixed CNV was driven by a different combination of vascular growth factors than the type 2 part. Unfortunately, we have no data to support this claim. As for the CNV maturation, based on the assessment of the OCTa, the type 1 part of the CNV was not fully matured at the start of the treatment while the type 2 part of the CNV was already well defined. In theory, this should have made the type 1 part of the CNV more susceptible to the ranibizumab treatment than the type 2 part, however, we observed the opposite. It is hard to assess whether the different distribution of ranibizumab and aflibercept in the retina could have played a role in our patient since both drugs were present in the choroidal and retinal vessel of tested monkey eyes.

The absence of response to anti-VEGF treatment can appear during the initial phase of treatment, or it can emerge later during the reactivation of the disease in patients who had previously responded well to treatment [6, 7]. The effectiveness of aflibercept in patients who do not respond to treatment with other anti-VEGF agents can be explained by its structure. It has a higher affinity for VEGF-A than either ranibizumab or bevacizumab and also binds other angiogenic factors such as VEGF-B and placental growth factor (PlGF) [16]. These properties should theoretically result in the greater effectiveness of aflibercept compared to ranibizumab and bevacizumab. Studies comparing ranibizumab against aflibercept showed comparable anatomical and functional results for both drugs in naïve patients, with aflibercept requiring fewer applications [3, 17].

In our patient, we observed an asymmetric response to ranibizumab in each portion of a mixed type 1 and type 2 CNV. Whereas the type 2 CNV reacted swiftly, type 1 CNV continued to grow despite intensive treatment with ranibizumab. High edema persisted in the macula and only slight improvement in BCVA was observed. Switching to treatment with aflibercept led to the reduction in size of type 1 CNV, to the reduction of macular edema and improvement in BCVA.

## Data Availability

All data generated or analyzed during this study are included in this manuscript.

## Abbreviations

CNV: Choroidal neovascular membrane
BCVA: Best-corrected visual acuity
ETDRS: Early Treatment Diabetic Retinopathy Study
OCTa: Optical coherence tomography angiography
OCT: Optical coherence tomography
nAMD: Neovascular age-related macular degeneration
Anti-VEGF: Anti-vascular endothelial growth factor
LE: Left eye
RE: Right eye
PED: Pigment epithelial detachment
CST: Central subfield thickness
RPE: Retinal pigment epithelium
PlGF: Placental growth factor
